# 17β-Estradiol Modulates Gene Expression in the Female Mouse Cerebral Cortex

**DOI:** 10.1371/journal.pone.0111975

**Published:** 2014-11-05

**Authors:** Gwendolyn I. Humphreys, Yvonne S. Ziegler, Ann M. Nardulli

**Affiliations:** Department of Molecular and Integrative Physiology, University of Illinois at Urbana-Champaign, Urbana, Illinois, United States of America; Florida International University, United States of America

## Abstract

17β-estradiol (E2) plays critical roles in a number of target tissues including the mammary gland, reproductive tract, bone, and brain. Although it is clear that E2 reduces inflammation and ischemia-induced damage in the cerebral cortex, the molecular mechanisms mediating the effects of E2 in this brain region are lacking. Thus, we examined the cortical transcriptome using a mouse model system. Female adult mice were ovariectomized and implanted with silastic tubing containing oil or E2. After 7 days, the cerebral cortices were dissected and RNA was isolated and analyzed using RNA-sequencing. Analysis of the transcriptomes of control and E2-treated animals revealed that E2 treatment significantly altered the transcript levels of 88 genes. These genes were associated with long term synaptic potentiation, myelination, phosphoprotein phosphatase activity, mitogen activated protein kinase, and phosphatidylinositol 3-kinase signaling. E2 also altered the expression of genes linked to lipid synthesis and metabolism, vasoconstriction and vasodilation, cell-cell communication, and histone modification. These results demonstrate the far-reaching and diverse effects of E2 in the cerebral cortex and provide valuable insight to begin to understand cortical processes that may fluctuate in a dynamic hormonal environment.

## Introduction

The effects of 17β-estradiol (E2) have been extensively studied in the female reproductive tract where it is required for reproductive competency. E2 also targets a variety of other tissues, including the mammary gland [1], bone [2], [3], cardiovasculature [4], and brain [5]. E2 plays several critical roles in brain development, such as influencing sexual dimorphism [6] and forming synapses [7]. In the cycling female, E2 is an important regulator of ovulation through its communication with the hypothalamus and pituitary [8], [9]. E2 can also act on brain regions not associated with reproduction and can influence pain perception, locomotion, and mood [10].

Numerous experiments have demonstrated that E2 protects the brain from a variety of insults [11]–[13]. For example, E2 protects neuroblastoma cells from H_2_O_2_ [14] and beta amyloid [15], [16] toxcicity. Additionally, E2 decreases cellular damage in neurons that have been treated with excitotoxic levels of glutamate [17] and hippocampal slice cultures that have been exposed to oxygen and glucose deprivation [18]. In vivo, E2 reduces inflammation [19], [20] and ischemia-induced damage [21], [22] and this protection is most evident in the cerebral cortex.

In addition to its neuroprotective effects, E2 modulates synaptic plasticity [23], influences neurotransmission [24], [25], and acts as a neurotrophin [26] to support brain homeostasis. These cumulative reports suggest that critical changes in gene expression in the brain are induced by E2. Although the cerebral cortex receives input from several brain regions and is essential for cognitive and executive functions [27], the mechanism by which E2 mediates its effects in the cerebral cortex are unclear. To better understand the molecular consequences of E2 in the cerebral cortex, we analyzed RNA sequencing (RNA-Seq) data from the cortices of oil- and E2- treated, ovariectomized female mice. This unbiased approach identified E2-regulated genes that provide insight into the multiple biological processes influenced by E2 treatment.

## Materials and Methods

### Animals and surgery

14 week old female C57BL/6J mice were obtained from Jackson Laboratory (Bar Harbor, ME) and maintained on a 12 hr light/dark schedule with access to water and food ad libitum. After 7 days, mice were anesthetized by inhalation of 4% isoflurane, bilaterally ovariectomized and then implanted subcutaneously with silastic tubing (0.062 in/0.125 in, inner/outer diameter, 1 in length; Dow Corning, Midland, MI) plugged at both ends with medical adhesive (Dow Corning). The silastic tubing, which remained in the mice for 7 days, contained either 35 µl of cottonseed oil or 35 µl of cottonseed oil with 180 µg/ml E2 and produced a low, physiological level of circulating E2 (∼25 pg/ml) [21], [28] that is equivalent to estrus levels in mice [29]. Ovariectomized mice were fed phytoestrogen-free chow and after 7 days, the mice were sacrificed, the brains were dissected, and cerebral cortices were harvested. This method of E2 treatment has been extensively used to demonstrate the anti-inflammatory and neuroprotective actions of E2 in the cerebral cortex [19], [21], [30], [31]. The protocol (#12014) for this study was approved and carried out in strict accordance with guidelines from the University of Illinois at Urbana-Champaign Institutional Animal Care and Use Committee and Division of Animal Resources. Analgesics were administered after surgery and all efforts were made to minimize suffering.

### RNA isolation

Total RNA was isolated from each cerebral cortex using Ambion RNAqueous according to the manufacturer’s protocol (Life Technologies, Grand Island, NY) and treated with Turbo DNA-free reagent (Ambion, Life Technologies, Austin, TX) to remove genomic DNA. RNA purity was assessed with native agarose gel electrophoresis and analysis of the 28S and 18S rRNA bands. RNA was of high purity, showed no degradation, and was free of DNA (Fig. S1).

### RNA-Sequencing

RNA-seq was completed at the W.M. Keck Center for Comparative and Functional Genomics at the University of Illinois Urbana-Champaign. The TruSeq RNA sample prep kit (Illumina, San Diego, CA) and 1 µg of total RNA were used to make poly-A selected and barcoded RNA-Seq libraries for each cortical sample. cDNA libraries were pooled and quantified using real-time PCR with the Library Quantification kit (Kapa Biosystems, Woburn, MA). The libraries were sequenced using 3 lanes for 101 cycles with 7 additional cycles for the index read on the Illumina HiSeq2000 according to the manufacturer’s instructions. The RNA-Seq libraries produced over 255 million reads with each individual sample having more than 29 million reads. The data was then used to generate Fastq files using Casava 1.8.2.

### RNA-Seq alignment and statistics

Sequences were aligned using TopHat v. 1.4.1 [32] and Bowtie 1.0 [33]. The genome sequence index was mm10 from UCSC (http://hgdownload.soe.ucsc.edu/downloads.html#mouse). Raw read counts were tabulated for each sample at the gene level using the GTF gene model file for mm10 from UCSC and htseq-count, from HTSeq v0.5.3p9 (http://www-huber.embl.de/users/anders/HTSeq/doc/index.html) using the default “exon” feature type and “gene_id” attribute.

The raw read counts were used in R 3.0.0 [34] for data pre-processing and statistical analysis using packages from Bioconductor [35] as indicated below. Data are available in the Array Express database under accession number E-MTAB-2762. Genes without 1 count per million (CPM) mapped reads in at least 4 samples, irrespective of group, were filtered out and 14,908 of the 37,482 genes passed this filter and were analyzed using edgeR 3.2.3 [36]. The raw count values were used in a negative binomial statistical model that accounted for the total library size for each sample and an extra TMM normalization factor for any biases due to changes in total RNA composition of the samples [37], [38]. Tests for the pairwise comparisons were pulled from the model and separately adjusted for multiple testing using the False Discovery Rate (FDR) method [39].

Comparable expression values were generated from read counts using voom normalized values [40]. The voom values were scaled to the standard deviation of the mean, hierarchically clustered, and displayed as heatmaps. Additional annotation information (gene names, descriptions) was obtained from Ensembl Genes 71, Mus musculus genes (GRCm38.p1) database using the Ensembl gene IDs provided in the GTF gene model file.

Cytoscape (Version 3.0.1) was used in conjunction with the plug-in ClueGO (Version 2.0.7) for network creation [41], [42]. KEGG [43], Reactome [44], and Gene Ontology (biological process) [45] databases were used within the program for network categorization. Over-representation (enrichment) was calculated in the program using a right-sided hypergeometric test and Bonferroni step-down method for multiple test correction.

Transcriptomine from the Nuclear Receptor Signaling Atlas website was used to determine previously identified E2-regulated genes. 17β-estradiol was selected as the ligand and >1.1 fold change in either direction with *p*<0.05 significance was selected for ‘CNS, all tissues and cell lines’ and ‘all tissues, all cell lines’ RNA sources.

### Real-Time PCR (RT-PCR)

RNA concentrations were measured and cDNA was synthesized using the iScript kit (Bio-Rad, Hercules, CA) as described by the manufacturer. cDNA was combined with iQ SYBR Green Supermix (Bio-Rad, Hercules, CA), and forward and reverse primers (0.9 µM) for receptor transporter protein 1, Rtp1, (5′-CTGCCCTGCCTTACACTTAC -3′ and 5′-TCACCTCCTCCATCTTCTCG -3′), macrophage galactose N-acetyl-galactosamine specific lectin 2, Mgl2, (5′- GTGACAAGAAAGGAGGAATG -3′ and 5′- GAGATGACCACCAGTAGC -3′), NLR-pyrin domain containing 3, Nlrp3, (5′- CCAAGGAGGAAGAAGAAGAG -3′ and 5′- AAGAGACCACGGCAGAAG -3′), fatty acid binding protein 7, Fabp7, (5′- GTGACCAAACCAACTGTGATTATC -3′ and 5′- TGTCTCCATCCAACCGAACC-3′), lysozyme 2, Lyz2, (5′- TGAAGACTCTCCTGACTC-3′ and 5′- ACGGTTGTAGTTTGTAGC -3′), succinate dehydrogenase complex, subunit A, flavoprotein, Sdha, (5′- GCTCATCGGTGTTGCTGTG-3′ and 5′-TTGCTCTTATTCGGTGTATGGAC -3′), aldolase A fructose-bisphosphate, Aldoa, (5′- GAGAACACCGAGGAGAAC-3′ and 5′-CCTTGGACTTGATAACTTGG -3′), or ribosomal protein L7, Rpl7, (5′- CGCACTGAGATTCGGATG-3′ and 5′-TTAATTGAAGCCTTGTTGAGC-3′). RT-PCR was carried out using a Bio-Rad iQ5 multicolor Real-Time PCR Detection System. Samples were run in triplicate with each primer set along with a standard curve. Ct values were normalized to Rpl7 using the delta-delta Ct method. Combined data are expressed as the mean ± SEM and Student’s t-test was used to detect significant (*p*<0.05) differences.

### Western Blotting

Extracts from cortical tissue were prepared using RIPA buffer (Thermo Scientific, Rockford, IL), protease inhibitors (Complete Mini, Roche, Mannheim, Germany) and phosphatase inhibitors (Phosphatase Inhibitor Cocktail Set III, Calbiochem, San Diego, CA). Samples were homogenized using a Pro Homogenizer (ProScientific Inc., Oxford, CT) and protein concentration was determined using the bicinchoninic acid (BCA) assay (ThermoScientific) with bovine serum albumin as a standard. 30 µg of protein was loaded per lane on a 4–12% gradient acrylamide gel. Proteins were transferred to a nitrocellulose membrane and probed with an anti-phosphorylated ERK (p44/p42 MAPK, #9101, Cell Signaling Technology, Danvers, MA) or an anti-ERK (p44/p42 MAPK, #4695, Cell Signaling Technologies) specific antibody. Western blots were imaged and quantitated using the Licor Odyssey Infrared Imaging System and pERK was normalized to total ERK. Combined data are expressed as the mean ± SEM and Student’s t-test was used to detect significant (*p*<0.05) differences in the levels of pERK.

### Results and Discussion

To identify E2-mediated alterations in the female cerebral cortical transcriptome, a comprehensive study was carried out using a mouse model system. Female mice were ovariectomized and implanted with silastic tubing containing oil alone or oil with E2, which produced a low, physiological level of E2 [21]. This method of E2 treatment has been used in several studies to demonstrate the anti-inflammatory and neuroprotective actions of E2 in the mouse cerebral cortex [19], [21], [30], [31]. After 7 days, brains were dissected and total RNA was isolated from the cerebral cortices. The isolated RNA was poly-A selected, converted to cDNA, and analyzed using RNA-Seq.

### E2 significantly altered gene expression in cerebral cortex

**Figure 1:**
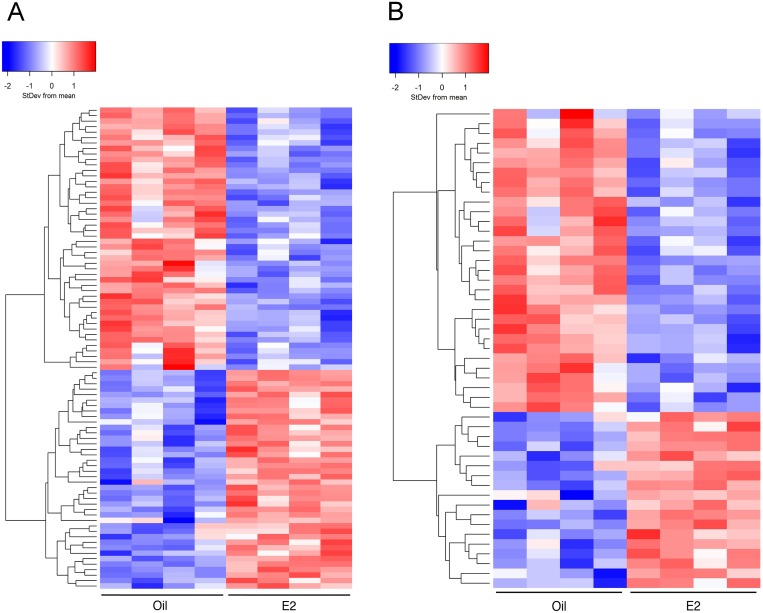
Heatmaps comparing cerebral cortices from oil- and E2-treated mice. Hierarchical clustering was used to visualize the transcript levels of (A) 88 genes that were significantly altered (FDR *p*<0.05) by E2 treatment or (B) 49 genes that were altered 1.2 fold or more by E2 treatment (FDR *p*<0.05). Each column represents cortical tissue from one mouse (8 mice total) and rows indicate genes. Colors symbolize increased (red) or decreased (blue) transcript levels.

E2 significantly (FDR *p*<0.05) altered the expression of 88 genes in the cerebral cortex (Table 1, Table S1). The expression of these genes is displayed on a heat map (Fig. 1A), where red indicates a significant increase and blue indicates a significant decrease in transcript levels. Interestingly, the number of genes decreased (48) by E2 treatment was slightly greater than the number of genes increased (40) by E2 treatment.

**Table 1:** 88 E2-responsive genes in the cerebral cortex.

Gene symbol	Description	FDR p-value	Fold change
2410137F16Rik	RIKEN cDNA 2410137F16 gene	0.020	1.7
Adcy9	adenylate cyclase 9	0.019	1.2
Agxt2l1	alanine-glyoxylate aminotransferase 2-like 1	3.1E-05	1.8
Ankrd33b	ankyrin repeat domain 33B	0.0071	1.2
Apln	apelin	0.0043	−1.3
Aqp4	aquaporin 4	0.00059	−1.3
Bcas1	breast carcinoma amplified sequence 1	3.1E-05	−1.3
Bhlhe40	basic helix-loop-helix family, member e40	0.0040	1.2
Btbd17	BTB (POZ) domain containing 17	0.020	−1.2
Cd82	CD82 antigen	0.0058	−1.3
Cdhr1	cadherin-related family member 1	0.038	−2.8
Cmtm5	CKLF-like MARVEL transmembrane domain containing 5	0.0038	−1.2
Cnp	2′,3′-cyclic nucleotide 3′ phosphodiesterase	0.010	−1.2
Col19a1	collagen, type XIX, alpha 1	0.048	1.3
Cpeb1	cytoplasmic polyadenylation element binding protein 1	0.030	1.1
Cryab	crystallin, alpha B	0.0017	−1.2
Dusp4	dual specificity phosphatase 4	0.038	1.3
Ednrb	endothelin receptor type B	0.011	−1.3
Elfn2	leucine rich repeat and fibronectin type III, extracellular 2	0.019	1.1
Elovl5	ELOVL family member 5, elongation of long chain fatty acids (yeast)	0.011	−1.2
Erbb4	v-erb-a erythroblastic leukemia viral oncogene homolog 4 (avian)	0.044	1.1
Fa2h	fatty acid 2-hydroxylase	0.041	−1.2
Fabp7	fatty acid binding protein 7, brain	0.001	−1.6
Fam107a	family with sequence similarity 107, member A	0.016	1.2
Fbxo33	F-box protein 33	0.026	1.1
Fcrls	Fc receptor-like S, scavenger receptor	0.030	1.2
Flnb	filamin, beta	0.046	1.1
Gdpd5	glycerophosphodiester phosphodiesterase domain containing 5	0.029	−1.2
Gfap	glial fibrillary acidic protein	0.0013	−1.3
Gja1	gap junction protein, alpha 1	0.044	−1.1
Gjc2	gap junction protein, gamma 2	0.00059	−1.3
Gltp	glycolipid transfer protein	0.023	−1.2
Gm20634	predicted gene 20634	0.019	−1.3
Gsn	gelsolin	0.0071	−1.2
Hadha	hydroxyacyl-Coenzyme A dehydrogenase, alpha subunit	0.019	−1.1
Herc1	hect domain and RCC1-like domain 1	0.039	1.1
Hist1h2bc	histone cluster 1, H2bc	0.029	−1.2
Hivep3	human immunodeficiency virus type I enhancer binding protein 3	0.010	1.2
Igfbp2	insulin-like growth factor binding protein 2	0.00090	−1.5
Igfbpl1	insulin-like growth factor binding protein-like 1	0.023	−2.3
Irs2	insulin receptor substrate 2	0.0022	1.3
Jam2	junction adhesion molecule 2	0.011	−1.2
Lyz2	lysozyme 2	0.0043	−1.7
Mag	myelin-associated glycoprotein	0.0017	−1.3
Mgl2	macrophage galactose N-acetyl-galactosamine specific lectin 2	2.6E-06	3.5
Mid1ip1	Mid1 interacting protein 1 (gastrulation specific G12-like (zebrafish))	0.038	−1.1
Mll1	myeloid/lymphoid or mixed-lineage leukemia 1	0.0020	1.2
Mvd	mevalonate (diphospho) decarboxylase	0.048	−1.2
Myoc	myocilin	0.0017	−1.4
Ndufa3	NADH dehydrogenase (ubiquinone) 1 alpha subcomplex, 3	0.022	−1.2
Nlrp3	NLR family, pyrin domain containing 3	0.0020	2.0
Nov	nephroblastoma overexpressed gene	0.010	1.3
Nuak1	NUAK family, SNF1-like kinase, 1	0.0013	1.2
Olfml3	olfactomedin-like 3	7.0E-06	−1.4
Pcnt	pericentrin (kendrin)	0.038	1.1
Pdgfb	platelet derived growth factor, B polypeptide	8.8E-08	1.3
Phf15	PHD finger protein 15	0.0013	1.2
Phf21b	PHD finger protein 21B	0.021	1.2
Plk2	polo-like kinase 2	0.022	1.1
Pllp	plasma membrane proteolipid	0.033	−1.2
Prdx6	peroxiredoxin 6	0.043	−1.1
Ptn	pleiotrophin	0.016	−1.2
Ptpn7	protein tyrosine phosphatase, non-receptor type 7	0.046	−1.7
Rtp1	receptor transporter protein 1	0.0043	2.8
Serpinb1a	serine (or cysteine) peptidase inhibitor, clade B, member 1a	0.011	−1.5
Siglec1	sialic acid binding Ig-like lectin 1, sialoadhesin	0.048	1.9
Slc13a3	solute carrier family 13 (sodium-dependent dicarboxylate transporter), member 3	0.014	−1.4
Slc25a1	solute carrier family 25 (mitochondrial carrier, citrate transporter), member 1	0.019	−1.2
Slc38a3	solute carrier family 38, member 3	0.048	−1.2
Sntb2	syntrophin, basic 2	0.012	1.3
Sowahb	sosondowah ankyrin repeat domain family member B	0.039	1.2
Spred2	sprouty-related, EVH1 domain containing 2	0.023	1.1
Srrm4	serine/arginine repetitive matrix 4	0.0017	1.2
Tcn2	transcobalamin 2	0.022	−1.2
Tet3	tet methylcytosine dioxygenase 3	0.016	1.1
Tfrc	transferrin receptor	0.0017	1.2
Tgfbr1	transforming growth factor, beta receptor I	7.1E-07	1.3
Tmem116	transmembrane protein 116	0.038	1.4
Top2a	topoisomerase (DNA) II alpha	0.022	−1.7
Trib2	tribbles homolog 2 (Drosophila)	0.017	1.2
Trp53inp2	transformation related protein 53 inducible nuclear protein 2	0.038	-1.1
Tsc1	tuberous sclerosis 1	0.017	1.1
Tst	thiosulfate sulfurtransferase, mitochondrial	0.0052	−1.2
Ugt8a	UDP galactosyltransferase 8A	0.038	−1.3
Unc5b	unc-5 homolog B (C. elegans)	0.021	−1.2
Vcam1	vascular cell adhesion molecule 1	0.016	−1.3
Vstm4	V-set and transmembrane domain containing 4	0.038	−1.4
Vwf	Von Willebrand factor homolog	3.1E-05	1.6

Of the 88 genes that responded to E2 treatment, 49 were altered 1.2 fold or more (FDR *p*<0.05, Fig. 1B). Again, the number of genes decreased (blue) by E2 treatment was greater than the number of genes increased (red) by E2 treatment suggesting that E2 is a more potent repressor than activator of gene expression in the cerebral cortex. However, both heatmaps demonstrated that E2 differentially regulates gene expression in the mouse cerebral cortex.

### Validation of E2 regulated genes

**Figure 2:**
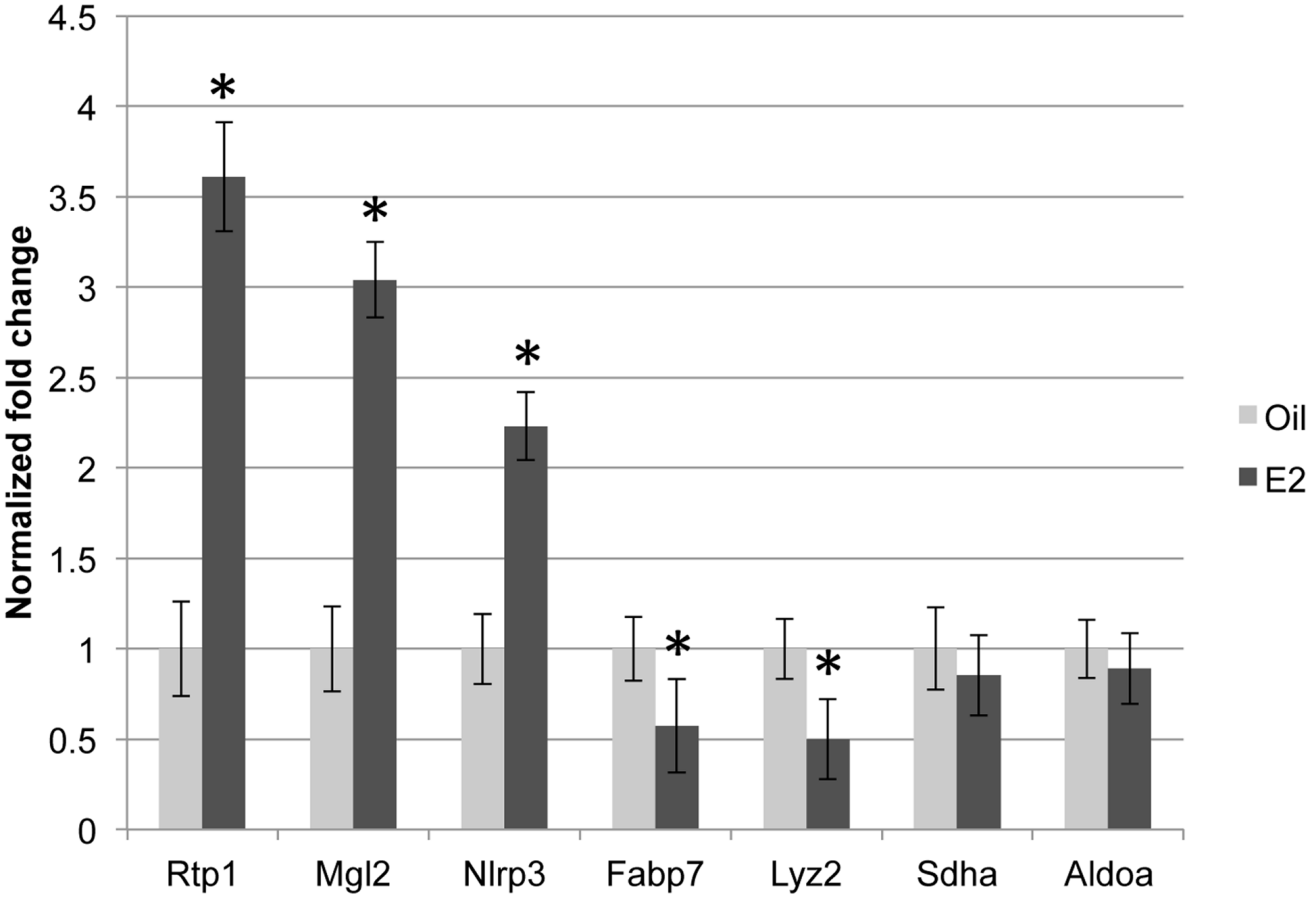
Validation of transcripts altered by E2 treatment. (A) Quantitative real-time PCR was conducted with gene-specific primers. The normalized fold change ± SEM was calculated using the delta-delta Ct method with Rpl7 as a control gene. The Student’s t-test was used to detect significant differences in oil- and E2- treated animals (4 animals/treatment, *p<0.05).

To validate the RNA-Seq data, we examined a subset of E2-regulated genes using RT-PCR analysis. In agreement with the RNA-Seq data, E2 increased Rtp1, Mgl2, and Nlrp3 expression, decreased Fabp7 and Lyz2 expression, and did not alter the expression of Sdha and Aldoa (Fig. 2, *p<0.05). Thus, RT-PCR analysis provided evidence of the accuracy of the RNA-Seq dataset.

### E2-regulated genes

We were interested in determining if any of the 88 genes that were significantly altered by E2 in the cerebral cortex have previously been reported as E2-responsive genes. Using the genome-wide expression profiling database Transcriptomine in the Nuclear Receptor Signaling Atlas, we found that each of the 88 genes except Rtp1, Gm20634, and 2410137FRik was listed as an E2-responsive gene in a variety of tissues or cultured cells [46], but only 5 genes, Aqp4, Bhlhe40, Ednrb, Erbb4 and Igfbp2, were designated as E2-responsive in the central nervous system. Two additional studies have reported that Gfap [47] and Slc13a3 [20] are E2-responsive in the brain. Thus, based on literature and database searches, the majority of the 88 genes identified in our dataset are novel, E2-regulated genes in the mouse cerebral cortex. Interestingly, earlier reports suggest that the gene expression profile of the hippocampus differs substantially from the gene expression profile of the cerebral cortex and that acute and chronic E2 treatments may differentially alter gene expression [48], [49].

The 10 genes that declined most significantly in response to E2 treatment are shown in Table 2. The largest E2-mediated decrease was observed in cadherin-related family member 1 (Cdhr1), which is a protocadherin in the cadherin superfamily, and functions as a calcium-dependent cell adhesion and signaling molecule [50]. The greatest E2-mediated increases in transcript levels are listed in Table 3. The expression of macrophage galactose N-acetyl-galactosamine specific lectin 2 (Mgl2), also referred to as CD301, was most significantly increased. The function of this gene in the cerebral cortex has not been described. However, microglia, the resident immune cells in the brain, often express multiple cluster of differentiation (CD) cell surface proteins [51]. Siglec1, another CD gene (CD169), is expressed on macrophages associated with the perivasculature in the rat brain [52]. These results suggest that E2 may be altering gene expression in immune cells.

**Table 2:** E2-regulated genes with the most decreased expression.

Gene symbol	Description	Fold decrease	FDR p value
Cdhr1	cadherin-related family member 1	−2.8	0.038
Igfbpl1	insulin-like growth factor binding protein-like 1	−2.3	0.023
Ptpn7	protein tyrosine phosphatase, non-receptor type 7	−1.7	0.046
Top2a	topoisomerase (DNA) II alpha	−1.7	0.022
Lyz2	lysozyme 2	−1.7	0.0043
Fabp7	fatty acid binding protein 7, brain	−1.6	0.0013
Serpinb1a	serine (or cysteine) peptidase inhibitor, clade B, member 1a	−1.5	0.011
Igfbp2	insulin-like growth factor binding protein 2	−1.5	0.0009
Olfml3	olfactomedin-like 3	−1.4	7.00E-06
Myoc	myocilin	−1.4	0.0017

**Table 3:** E2-regulated genes with the most increased expression.

Gene symbol	Description	Fold increase	FDR p value
Mgl2	macrophage galactose N-acetyl-galactosamine specific lectin 2	3.5	2.60E-06
Rtp1	receptor transporter protein 1	2.8	0.0043
Nlrp3	NLR family, pyrin domain containing 3	2	0.002
Siglec1	sialic acid binding Ig-like lectin 1, sialoadhesin	1.9	0.048
Agxt2l1	alanine-glyoxylate aminotransferase 2-like 1	1.8	3.10E-05
2410137F16Rik	RIKEN cDNA 2410137F16 gene	1.7	0.02
Vwf	Von Willebrand factor homolog	1.6	3.10E-05
Tmem116	transmembrane protein 116	1.4	0.038
Pdgfb	platelet derived growth factor, B polypeptide	1.3	8.80E-08
Sntb2	syntrophin, basic 2	1.3	0.012

### Networks of E2-responsive biological processes and pathways

**Figure 3:**
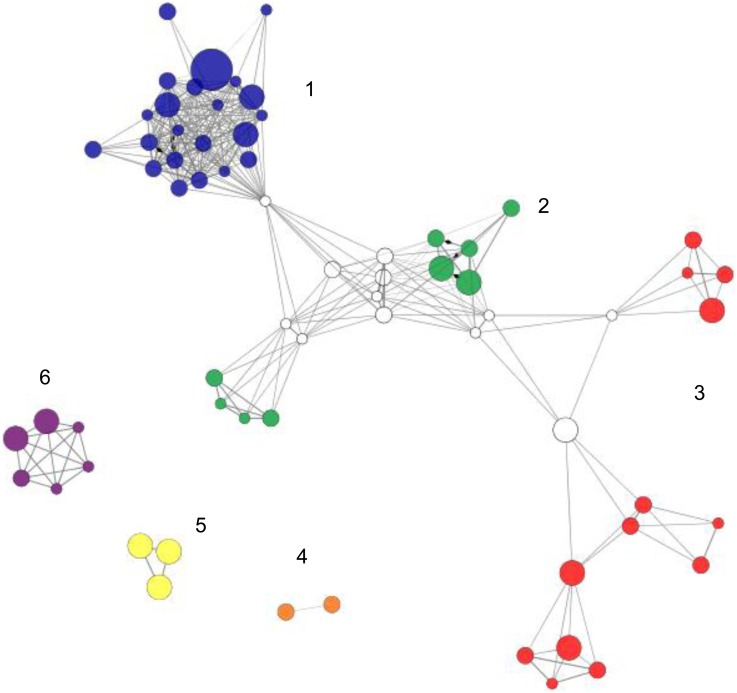
Networks of E2-regulated genes. ClueGO analysis classified the 88 E2-regulated genes into 6 networks. White nodes indicate that a biological process is associated with two networks. Node size indicates the statistical significance of the biological process represented. Thus, larger nodes indicate greater statistical significance.

To begin to understand how E2 regulates the cerebral cortical transcriptome, the 88 E2-responsive genes were uploaded to ClueGO [42] to identify networks of biological processes and pathways that are altered by E2 treatment (Fig. 3 and Table 4). The nodes (filled circles) represent biological processes or pathways associated with the E2-regulated genes based on gene ontology terms [45], Reactome [44] and KEGG [43] databases. Related nodes are clustered together in color-coded networks and all of the nodes in a network are the same color. However, a node can participate in two networks and those nodes are white. The size of the node reflects the level of statistical significance of each of the E2-regulated biological processes or pathways. Thus larger nodes have increased statistical significance.

**Table 4:** E2-responsive networks and associated genes.

Biological process GO term or Pathway	Genes
**Network 1**	
Regulation of centrosome cycle	Gja1, Plk2
Inactivation of MAPK activity	Dusp4, Spred2
Cell-cell junction assembly	Gja1, Ugt8a
Regulation of tissue remodeling	Gja1, Tfrc
Regulation of mRNA splicing, via spliceosome	Gja1, Srrm4
Carbohydrate derivative transport	Gja1, Gltp
Regulation of signal transduction by p53 class mediator	Gja1, Spred2
Long-term potentiation	Gfap, Plk2
Regulation of cell junction assembly	Gja1, Tsc1
Lens development in camera-type eye	Cryab, Gja1, Tgfrb1
Regulation of phosphoprotein phosphatase activity	Nuak1, Tsc1
rRNA transport	Gja1, Tsc1, Tst
**Network 2**	
Cerebellum morphogenesis	Herc1, Pcnt
Vasoconstriction	Apln, Ednrb, Pdgfb
Vasodilation	Apln, Cnp, Pdgfb
**Network 3**	
Fatty acid elongation	Elovl5, Hadha
Triglyceride biosynthesis	Elovl5, Slc25a1
Histone H4 acetylation	Mll1, Phf15
Histone H3-K4 methylation	Mll1, Tet3
Regulation of ligase activity	Mid1ip1, Trib2
Lysine degradation	Hadha, Mll1
Negative regulation of protein complex disassembly	Gsn, Mid1ip1
**Network 4**	
Response to estradiol stimulus	Aqp4, Cryab, Igfbp2
Vasopressin-regulated water reabsorption	Adcy9, Aqp4
**Network 5**	
Myelination	Fa2h, Pllp, Tsc1, Ugt8a
**Network 6**	
Meiotic chromosome separation	Bhlhe40, Top2a
**Network 1 and 2**	
Olfactory lobe development	Erbb4, Pcnt
Regulation of phosphatidylinositol 3-kinase activity	Erbb4, Pdgfb
Tissue regeneration	Erbb4, Gja1
Regulation of lipid kinase activity	Erbb4, Pdgfb
**Network 2 and 3**	
Positive regulation of fatty acid metabolic process	Irs2, Mid1ip1
Fatty acid beta oxidation	Hadha, Irs2
Regulation of polysaccharide metabolic process	Irs2, Pdgfb

The largest network is comprised of 22 nodes (Fig. 3, blue nodes) and the 14 genes associated with these nodes are listed in Table 4. Genes associated with this network include glial fibrillary acidic protein (Gfap) and polo-like kinase 1 (Plk2), which play a role in long term synaptic potentiation. Synaptic plasticity, learning, and memory are linked to long term synaptic potentiation. Dual specificity phosphatase 4 (Dusp4) and sprouty-related, EVH1 domain containing 2 (Spred2) are associated with the MAPK pathway, which is important in synaptic plasticity and also plays a role in cell signaling [53]. NUAK family, SNF 1-like kinase (Nuak1) and tuberous sclerosis 1 (Tsc1) are associated with phosphoprotein phosphatase regulation and may be contributing to modulation of protein phosphorylation. Taken together, these results suggest that E2 is affecting important signal integration pathways in the cerebral cortex.

Unique to network 2 (green) were biological processes involved in vasoconstriction and vasodilation which included platelet derived growth factor B (Pdgfb) and endothelin receptor type B (Ednrb). Networks 1 and 2 shared genes involved in PI3K activity such as v-erb-a erythroblastic leukemia viral oncogene homolog 4 (Erbb4) and Pdgfb. The PI3K pathway is important for E2 signaling and inhibition of this pathway blocks downstream ERK activation by E2 in cortical neuron cultures [54]. Fatty acid synthesis is a critical function in the brain, which contains the second highest level of lipids in the body after adipose tissue [55]. Fatty acid metabolic processes were associated with insulin receptor substrate 2 (Irs2) and Mid1 interacting protein 1 (Mid1ip1) in network 3. Mid1ip1 enhances fatty acid synthesis and its overexpression in the liver causes triglyceride accumulation [56]. Irs2 is critical in regulating brain size, since the brains of Irs2 null mice are reduced by ∼50% due to decreased neuronal proliferation [57].

Pathways involved in lipid synthesis were also present in network 3 (red) and included genes ELOVL family member 5, elongation of long chain fatty acids (Elovl5) and hydroxyacyl-Coenzyme A dehydrogenase/3-ketoacyl-coenzyme A thiolase/enoyl-Coenzyme A hydratase alpha subunit (Hadha) and the solute carrier 25 (Slc25a1). Regulation of ligase activity was associated with Mid1ip1 and tribbles homolog 2 (Trib2). In addition to its role in fatty acid synthesis, Mid1ip1 interacts with Mid1, a ubiquitin ligase and microtubule associated protein [58], [59]. Trib2 functions as an adaptor for protein degradation through interactions with the E3-ubiquitin ligase Cop1 [60]. Gelsolin (Gsn), which encodes an actin binding protein involved in signaling and cytoskeletal remodeling [61] is associated with negative regulation of protein complex disassembly.

Three networks (4–6) were not connected to any of the other networks. Genes previously reported to be estrogen responsive in various cell types were included in network 4 (orange). The genes in this group included crystallin alpha b (Cryab), aquaporin 4 (Aqp4), and insulin-like growth factor binding protein 2 (Igfbp2), which have been reported as E2-responsive genes in the mouse uterus [62], cultured rat cortical neurons [63], and rat hippocampal tissue, respectively [64], [65]. Myelin is essential for proper nerve conduction [66] and network 5 (yellow) contained several genes associated with myelination. Fatty acid 2-hydroxylase (Fa2h), plasma membrane proteolipid (Pllp), UDP galactosyltransferase 8A (Ugt8a), and tuberous sclerosis 1 (Tst1) have been associated with oligodendrocytes, which produce myelin. Network 6 (purple) included DNA topoisomerase 2A (Top2a) and basic helix loop helix family, member 40 (Bhlhe40) which are both involved in chromosome separation. However, Top2a has also been reported to be expressed in cortical neurons [67] and Bhlhe40, also known as Stra13, has been implicated in neuronal differentiation [68]. Thus the roles of these genes extend beyond chromosomal separation.

The network analysis provided insight into the diverse array of functions that were affected by E2 treatment. However, this analysis is constrained by existing information in the databases used, which consequently did not include all of the 88 genes we identified. Thus we utilized literature searches to provide a more comprehensive picture of the pathways and processes that were affected by E2 treatment.

### Signaling Pathways

**Figure 4:**
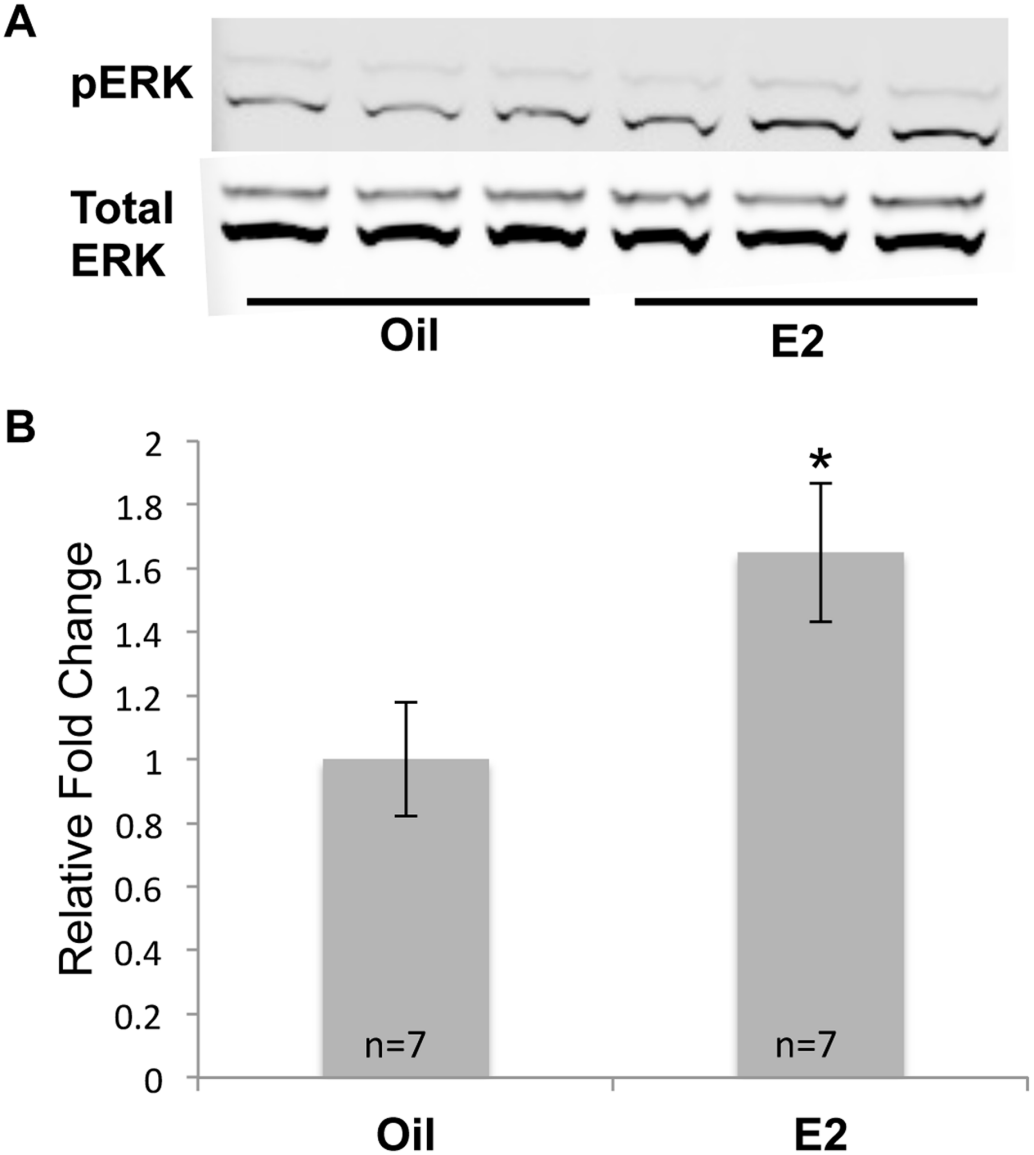
E2 increases pERK protein levels. (A)Western blot analysis was used to monitor pERK and total ERK levels in the cortices of mice that had been treated with oil or E2 for 7 days. (B) pERK values were normalized to total ERK and are displayed as the normalized fold change ± SEM. The Student’s t-test was used to detect significant differences in oil- and E2- treated animals (*p<0.05). The number of animals in each treatment group is indicated at the base of each bar.

The MAP kinase pathway plays a critical role in neuronal plasticity and survival [53] and E2 has been implicated in inducing rapid signaling through this pathway in neuroblastoma cells, primary cortical neurons, cortical explants, and the cerebral cortex in vivo [54], [69]–[71]. To determine whether MAP kinase signaling was activated after longer E2 treatment, we examined the level of phosphorylated extracellular regulated kinase (pERK) in the cortices of mice that had been treated with oil or E2 for 7 days. In fact, the level of pERK was significantly increased in the E2-treated animals (Fig. 4) demonstrating that E2 modulation of pERK and MAP kinase signaling is not limited to acute exposures (5–30 min), but is still enhanced after a longer treatment time.

We identified several E2-regulated genes in the cerebral cortex that modulate the MAP kinase signaling pathway. Dual specificity phosphatase 4 (Dusp4) dephosphorylates ERK [72] and is increased by E2 in breast cancer cells [73]. Thus the E2-mediated increase in Dusp4 expression could lead to decreased ERK phosphorylation. Interestingly, expression of protein tyrosine phosphatase, non-receptor type 7 (Ptpn7) was decreased with E2 treatment and may also be involved in ERK dephosphorylation [74]. In addition, expression of a repressor of MAP kinase activity, Spred2, [75] was modestly increased with E2 treatment. The fine-tuned expression of these genes by E2 highlights the balance that is needed between phosphorylation and dephosphorylation in the MAP kinase pathway [74]. Dusp4, Ptpn7 and Spred2 are novel, E2-responsive modulators of MAP kinase in the cerebral cortex.

### Cerebral cortex microvasculature

The brain is one of the most highly perfused organs in the body [76]. Proper brain function relies on maintenance of an extensive network of capillaries that form the cerebral microvasculature, which supplies oxygen and nutrients to meet the demands of this highly metabolic tissue. The microvasculature is comprised of endothelial cells surrounded by an extracellular matrix and a variety of cell types including neurons, astrocytes, microglia, and pericytes [51]. This complex network is referred to as the “neurovascular unit” since cooperation between these cells is necessary to maintain microvascular function [77]. Tight junctions between endothelial cells, together with the surrounding astrocyte end feet and pericytes form the blood-brain barrier (BBB), which carefully regulates the exchange of nutrients, water and other molecules. A dysfunctional BBB can lead to neurodegeneration and is the hallmark of several brain injuries [78].

Previous studies have shown that E2 decreases BBB permeability and thereby limits ischemic damage [79]. We identified several genes involved in BBB regulation that were altered by E2 treatment. Pdgfb transcript levels were increased by E2 treatment. Since Pdgfb binds to the Pdgfb receptor on pericytes [80] and mice with low Pdgfb levels have a dysfunctional BBB [81], Pdgfb is necessary for pericyte proliferation and maintenance [82], [83] and a functional neurovascular unit. Therefore, E2 may be acting to stimulate synthesis of Pdgfb in pericytes and endothelial cells, which could enhance autocrine and paracrine signaling to support BBB function.

E2 decreased the expression of Aqp4, a water transporter present on astrocyte end feet. These findings are in agreement with a previous report which indicated that E2 decreases Aqp4 expression and reduces hypoxia-induced swelling of rat cortical astrocytes in vitro [63]. E2 also decreased the expression of two solute carriers, Slc13a3, a sodium decarboxylate cotransporter and Slc38a3, an amino acid transporter, that have been associated with the BBB [84]. Together, the E2 mediated reduction in Aqp4, Slc13a3, and Slc38a3 could alter the exchange of water and solutes at the BBB and help to maintain fluid balance and homeostasis in the brain.

Von Willebrand Factor (Vwf) was increased by E2 treatment. Vwf is highly expressed in endothelial cells of brain microvasculature [85] and Vwf-null mice have increased damage compared to their wild-type counterparts after exposure to hypoxia and reoxygenation [86], suggesting that this factor is necessary for BBB adaptability and may help the brain to recover from an hypoxic event.

E2 has antinflammatory effects on the vasculature [87]. We found that E2 treatment decreased Vcam1 expression in the cerebral cortex. Vcam1 attracts leukocytes and monocytes to inflamed endothelial cells [88]. It has been proposed that E2 may decrease inflammation of endothelial cell cultures that have been subjected to an inflammatory agent by decreasing Vcam1 expression [89]. In addition, the E2 induced increase of Tgfb receptor 1 (Tgfbr1) may increase the sensitivity of Tgfb signaling, thus reducing inflammation [90]. The combined effects of E2 on maintaining the BBB (Pdgfb, Aqp4, Slc13a3, Slc38a3, Vwf) and reducing inflammation (Vcam1, Tgfbr1) could help to protect the cerebral cortex from injury.

### Oligodendrocytes and Myelin

Oligodendrocytes insulate neuronal axons by extending processes that produce lipid rich myelin. Myelin ensheathment of axons is important for nerve conduction and the loss of myelin leads to neurodegeneration. We were surprised at the number of oligodendrocyte-associated genes that were E2 responsive. Expression of myocillin (Myoc), myelin-associated glycoprotein (Mag), UDP galactosyltransferase 8a (Ugt8a), fatty acid 2-hydrolase (Fa2h), 2′, 3′-cyclic nucleotide 3′ phosphodiesterase (Cnp), CKLF-like MARVEL transmembrane domain containing 5 (Cmtm5) and plasma membrane proteolipid (Pllp) were all decreased by E2 treatment. A previous study found that turnover of oligodendrocytes in female rodents was increased and that Cnp protein expression was less than in males [91]. The decreased expression of these genes could indicate that E2 increases oligodendrocyte turnover rates in the cerebral cortex as well. However, much remains to be learned about the molecular consequences of decreased expression in this subset of oligodendrocyte- associated genes.

### Neurite extension

Neurite outgrowth is important for neuronal development, communication and function [92]. Impairment of neurite extension is associated with aging and neurodegeneration [93]. E2 can increase neurite extension in a variety of brain regions through several pathways including growth factor signaling, PI3K, and MAP kinase pathways [94]. We identified several genes involved in neurite extension that were altered by E2 treatment. E2 treatment modestly increased expression of Erbb4, which encodes a transmembrane protein that binds to neuregulin 1 (Nrg1). Erbb4-Nrg1signaling enhances neurite outgrowth through activation of the PI3K and MAP kinase pathways [95]. The E2-mediated increase in Erbb4 in the cerebral cortex could enhance neuritogenesis.

The expression of Igfbpl1 and Igfbp2 was decreased by E2 treatment. Igfbpl1 and Igfbp2 bind and sequester growth factors such as Igf1 [96]. Although Igfbpl1 is expressed in the developing mouse forebrain [97], the role of Igfbpl1 in the cerebral cortex has not been examined. In breast cancer cells, a decrease in Igfbpl1 has been associated with an increase in Igf1 levels [98]. E2 decreases Igfbp2 in the hippocampus [64] which can modulate Igf1 signaling pathways [99]. Moreover, Igf1 and E2 act synergistically to promote neurite outgrowth [100]. The E2-mediated decrease in expression of both Igfbpl1 and Igfbp2 could potentially allow growth factors such as Igf1 to circulate and promote neurite extension.

Glial fibrillary acidic protein (Gfap), an intermediate filament protein specifically expressed by astrocytes, can inhibit neurite outgrowth [47], [101]. The E2-mediated decrease in expression of Gfap in our studies suggests that E2 supports neurite extension, and could prevent an age-related increase in Gfap expression.

### Overall Implications

E2 alters gene expression through classical pathways that involve binding of the E2-occupied receptor to DNA. E2 can also act through non-classical pathways, by activation of membrane-associated proteins and rapid signaling pathways such as MAP kinase and PI3K, both of which have been shown to be important in the brain [102], [103]. It has been suggested that cross-talk occurs amongst the various E2 signaling pathways [104], [105] and that the cumulative E2-activation of several pathways may be required for effective E2-mediated neuroprotection [102].

Our study reflects the complex nature of E2 action and suggests that multiple signaling pathways in the cerebral cortex converge to orchestrate a diverse array of molecular events including those related to cerebrovascular function, neurite outgrowth, and brain homeostasis. The molecular impact of E2 treatment has particular relevance when considering the physiological consequences of menopause and estrogen replacement therapy. Further understanding of these events may provide insight into mechanisms responsible for estrogen-mediated gene expression and promote development of targeted treatments that support brain homeostasis.

## Supporting Information

Figure S1 **RNA gel demonstrating intact RNA samples.** Native agarose gel electrophoresis was used to resolve the intact 28s and 18s rRNA bands. 2 µg of RNA were run per lane.
(PDF)

Table S1 **88 E2-responsive genes in the cerebral cortex.**
(XLSX)
