# Supplementary material for: 17β-Estradiol Modulates Gene Expression in the Female Mouse Cerebral Cortex
Source: PLoS One. 2014 Nov 5;9(11):e111975. doi: 10.1371/journal.pone.0111975 (PMC4221195; doi:10.1371/journal.pone.0111975)
Supplement: Figure S1 — RNA gel demonstrating intact RNA samples. Native agarose gel electrophoresis was used to resolve the intact 28s and 18s rRNA bands. 2 µg of RNA were run per lane. (PDF) [file pone.0111975.s001.pdf]

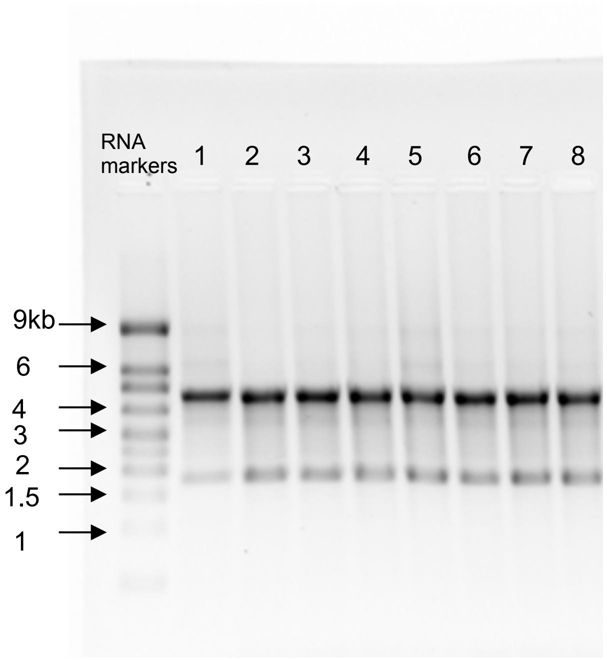

Figure S1. RNA gel demonstrating intact RNA samples. Native agarose gel electrophoresis was used to resolve the intact 28s and 18s rRNA bands. 2ug of RNA were run per lane.
